# Dermoscopic Aspects of Cutaneous Adverse Drug Reactions

**DOI:** 10.5826/dpc.1101a136

**Published:** 2021-01-29

**Authors:** Gabriela Rossi, André da Silva Cartell, Renato Marchiori Bakos

**Affiliations:** 1 Department of Dermatology, Universidade Federal do Rio Grande do Sul, (UFRGS), Porto Alegre, Brazil; 2 Department of Pathology, Hospital de Clínicas de Porto Alegre (HCPA) & Universidade Federal do Rio Grande do Sul (UFRGS), Porto Alegre, Brazil; 3 Department of Dermatology, Hospital de Clínicas de Porto Alegre (HCPA) & Universidade Federal do Rio Grande do Sul (UFRGS), Porto Alegre, Brazil

**Keywords:** cutaneous adverse drug reactions, dermoscopy, drug eruptions, dermoscopic patterns

## Abstract

**Background:**

Little is known about the dermoscopic evaluation of cutaneous adverse drug reactions (CADRs).

**Objectives:**

To evaluate the dermoscopic patterns of CADRs and identify those associated with severe cutaneous adverse reactions to drugs (SCARDs).

**Patients and Methods:**

Patients included in this study from May 2015 to April 2016 had presented with CADRs. CADR presentation and classification were based on standard criteria. SCARDs included Stevens-Johnson syndrome (SJS), toxic epidermal necrolysis (TEN), overlap SJS/TEN, drug reaction with eosinophilia and systemic symptoms (DRESS), and acute generalized exanthematous pustulosis (AGEP). The dermoscopic features of CADRs were described and compared according to the severity of the reactions.

**Results:**

Sixty-nine patients were included. Sixteen patients (23.2%) presented SCARDs. The main dermoscopic findings in SJS, overlap SJS/TEN and TEN were black dots or necrotic areas (100%). Erosion [respectively, 4/6 (66.7%), 3/3 (100%) and 1/1 (100%)], necrotic borders [respectively, 4/6 (66.7%), 3/3 (100%) and 1/1, (100%)] and epidermal detachment [respectively, 5/6 (83.3%); 2/3 (66.7%) and 1/1 (100%)] were also common among these reactions. Erythema and purpuric dots were the main dermoscopic findings [respectively, 5/6 (83.3%) and 4/6 (66.7%)] in DRESS. In non-severe reactions, the most prevalent structures were erythema and purpura in exanthema [respectively, 31/33 (93.9%) and 24/33 (72.7%)] and erythema and vascular structures in urticarial reactions [respectively, 6/6 (100%) and 3/6 (50%)]. Black dots or necrotic areas, epidermal detachment, necrotic borders and erosion were highly associated with SCARDs (P < 0.001).

**Conclusions:**

Dermoscopy improves clinical recognition of SCARDs.

## Introduction

Adverse drug reactions (ADRs) cause disability, prolonged hospitalization, increased health care costs, and even mortality. According to one meta-analysis, severe ADRs contributed to 6.7% of hospitalized admissions in USA [1]. Cutaneous adverse drug reactions (CADRs) are the most common type of ADRs [2,3]. ADRs may include any changes in the skin, its appendages or mucous membranes related to drug eruption and may be part of a systemic reaction [4–7]. Severe cutaneous adverse reactions to drugs (SCARDs) include Stevens-Johnson syndrome (SJS), toxic epidermal necrolysis (TEN), drug hypersensitivity syndrome or drug reaction with eosinophilia and systemic symptoms (DRESS), and acute generalized exanthematous pustulosis (AGEP) [8,9].

The incidence of CADRs among hospitalized patients ranges from 0.14% to 3.3% and from 0.14% to 1.05% in outpatients [6,10–16]. The most common CADR is morbilliform exanthema, followed by urticarial or fixed drug eruption [10–12,16–20]. SCARDs represent 0.13% to 16.5% [11,15,18,21,22]. The drugs most frequently involved are, in general, antibiotic agents, analgesics, nonsteroidal anti-inflammatory drugs (NSAIDs), contrast media, anticonvulsants, and chemotherapy [6,10,11,13,16–20,22,23].

CADRs most often have benign outcomes and are self-limited as long as the causative drug is stopped. Some cases, however, can be severe and even life-threatening [8,16,21,24,25]. Signs and symptoms that should alert the clinician to the possibility of a SCARD include mucosal involvement, extensive purpura, fever, blisters or epidermal detachment, facial edema, confluent erythema, painful eyes or skin, grayish skin lesions, marked eosinophilia, and lymph-adenopathy [26–28]. These changes do not always occur early, making it necessary to identify other findings that allow the detection of warning signs.

Dermoscopy is a safe in vivo diagnostic tool that improves diagnostic accuracy in several tumors. It has also been used also for the recognition of several skin diseases in general dermatology [29,30]. There are only a few reports regarding the application of dermoscopy in the detection of CADRs. We aimed to evaluate the dermoscopic findings of distinct CADRs and to determine the dermoscopic patterns of severe reactions.

## Patients and Methods

The protocol was approved by our local Ethics Committee and conducted in accordance with the Declaration of Helsinki. All patients were provided written informed consent before study procedures were initiated.

This prospective study was conducted at the University hospital where we recruited consecutive inpatients with suspected CADR that were evaluated by the Department of Dermatology. Patients of all ages presenting with cutaneous lesions following intake of any drugs were included in the study. The attending dermatologist made the diagnosis. Exclusion criteria were infectious exanthemas and other rashes associated with systemic or cutaneous diseases.

The presentation patterns of CADRs and their classification as severe (SJS, TEN, DRESS, AGEP, overlap SJS/TEN) or non-severe (all other presentations) were based on standard criteria, morphology, and the presence of severity markers. Demographic data, drugs used prior to the adverse reaction, concurrent medical conditions, concomitant medications, past history of drug allergy, and routine blood test results were collected. Histopathology was performed in all equivocal cases.

All patients had their skin reactions evaluated by dermoscopy. We used a non-polarized light hand-held dermoscope (DermLite II Hybrid M, 3Gen, San Juan Capistrano, CA) attached to a video camera. The presence of the following dermoscopic structures was evaluated: erythema, black dots or necrotic areas, necrotic borders, purpuric dots, vascular structures, scales, erosion, and epidermal detachment. We defined erythema as a diffuse occurrence of erythema in the lesion. Black dots or necrotic areas correlated to the presence of an area of diffuse tiny black dots. Epidermal detachment was defined as an eroded area in which the epidermis was detached, exposing the dermis, surrounded by dark brown or black necrotic lines marking the border.

Initially, the prevalence of the dermoscopic structures among the distinct SCARDs and CADRs was described. Then we sought to compare the presence of distinct dermoscopic structures between severe and non-severe presentations.

Quantitative variables with symmetric distribution were reported as mean with standard deviation and the Student t test was used. Quantitative variables with asymmetric distribution were reported as median and interquartile interval, and Mann-Whitney test was used. Categorical variables were reported as range and Fisher’s exact or the chi-square test was used. A P < 0.05 indicated statistical significance. The statistical analysis was performed using IBM SPSS Statistics 20.0.

## Results

Sixty-nine patients diagnosed with CADRs were enrolled in this study from May 2015 to April 2016. The mean age was 45.9 (SD 22.4 years, range from 0 to 89). Most were female (56.5%). All patients had some concurrent clinical condition. Hypertension, cancer, and HIV infection were the main comorbidities (36.2%, 31.9%, and 17.4% respectively).

The drugs most commonly implicated with CADRs were antibiotics (n = 20, 29.0%), anticonvulsants (n = 10, 14.5%) and dipyrone (n = 5, 7.2%). In 23 patients (33.3%) the drug related to CADR was undetermined.

The patients’ skin types according to Fitzpatrick classification were type II in 12 (17.4%), type III in 36 (52.2%), type IV in 16 (23.2%), type V in 3 (4.3%), and type VI in 2 (2.9%).

Thirty-three patients (47.8%) were diagnosed with exanthema, which was the most frequent clinical presentation. Other non-severe presentations were urticaria (6 patients; 8.7%), erythema multiforme (5 patients; 7.2%), cutaneous vasculitis (4 patients; 5.8%), erythroderma (2 patients, 2.9%), photosensitized eczematous reactions (2 patients; 2.9%), and fixed drug eruption (1 patient, 1.4%). Sixteen patients (23.2%) presented severe reactions. These were comprised of 6 patients with DRESS (8.7%), 6 patients with SJS (8.7%), 3 patients with overlap SJS/TEN (4.3%), and 1 patient with TEN (1.4%).

The median time from the onset of the cutaneous reaction to dermoscopic evaluation was 5 days (interquartile interval 25–75: 2.5–9.5 days).

Descriptive results of the dermoscopic analysis in different presentations of CADRs are presented in Table 1. We could observe that black dots or necrotic areas, epidermal detachment and erythema were the most prevalent dermoscopic structures in SJS, occurring respectively in 6 (100%), 5 (83.3%), and 5 (83.3%) of the cases. In the cases of overlap SJS/TEN and TEN cases, we also found black dots and epidermal detachment, in addition to necrotic borders and erosion. Erythema and purpuric dots were the most common dermoscopic structures in DRESS, present in 5 (83.3%) and 4 (66.7%) patients, respectively. Regarding the dermoscopic findings of the major non-severe reactions, we found that exanthemas presented erythema and purpuric dots as major features in, respectively, 31 (93.9%) and 24 (72.7%) of the patients. Erythema multiforme lesions also showed erythema and purpuric dots as the most frequent structures, occurring in 4 (80%) of the patients. Urticarial reactions most frequently included erythema and vascular structures, present in 6 (100%) and 3 (50%) of the cases, respectively.

Comparing SCARDs with non-SCARDS, we observed that the presence of black dots, epidermal detachment, necrotic borders, erosion and scales correlated significantly with the occurrence of the severe reactions (Table 2).

## Discussion

The diagnosis and classification of CADRs are based on clinical history, lesion features, and laboratory evaluation. Our study described the dermoscopic patterns of different presentations of CADRs and correlated them with severity, suggesting that dermoscopy might provide useful information for the evaluation of such patients.

**Figure 1:**
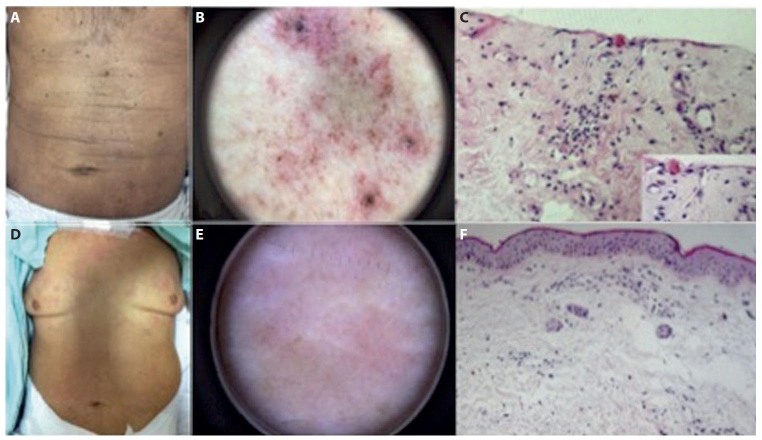
Early manifestations of drug reactions in skin. (A) Clinical image of a patient with Stevens-Johnson syndrome on day 3 after symptom onset; (B) dermoscopic image showing slightly scattered black dots; and (C) histopathologic image showing a necrolytic epidermis and a necrotic keratinocytes. (D) Clinical image of a patient with an exanthematous reaction; (E) dermoscopic image showing only diffuse erythema; and (F) histopathologic image showing perivascular inflammatory infiltrate and ectatic vessels.

**Figure 2:**
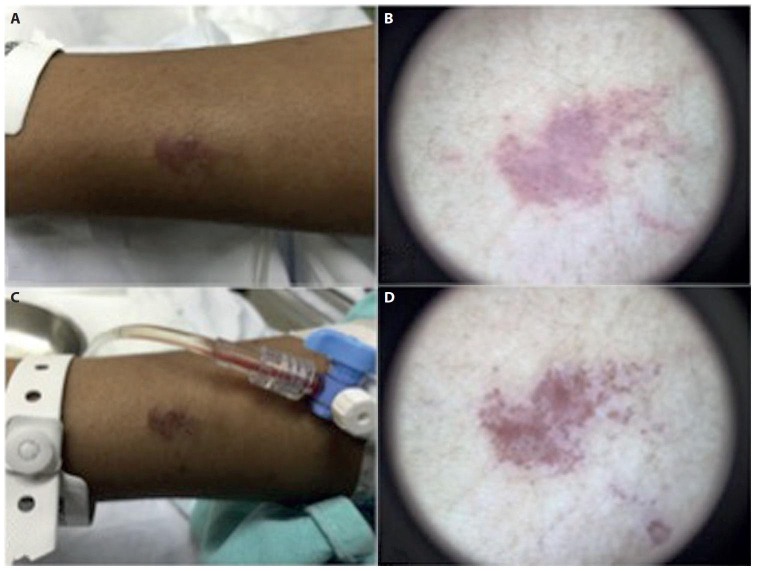
Clinical and dermoscopic images of a patient with Stevens-Johnson syndrome. On baseline evaluation (A) erythema and few tiny black dots are visualized on (B) dermoscopy. (C) After 3 days of follow-up, (D) black dots are diffusely distributed over the lesion.

**Figure 3:**
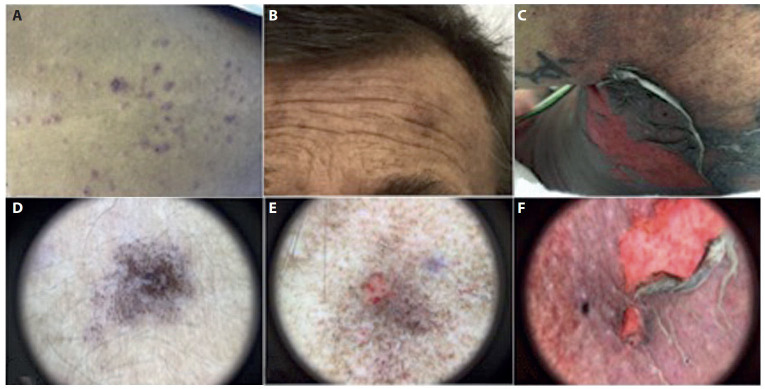
(A, B, C) Stevens-Johnson syndrome cases showing distinct clinical features. Dermoscopy demonstrating distinct structures: (D and E) black dots; (E) necrotic borders and erosion; and (F) epidermal detachment. While dermoscopy may lead to early suspicion of the skin reactions in the first 2 patients (A and B), the third patient (C) is clearly diagnosed by his clinical scenario. Nevertheless, the dermoscopic structures are also more pronounced (F) (original magnification, ×10).

Dermoscopically, SCARDs like SJS, overlap SJS/TEN and TEN showed a predominant pattern of black dots or necrotic areas, necrotic borders, erosion and detachment (Figures 1–3). These dermoscopic findings are similar to what might be generally observed in the clinical setting, and it is characterized by dark red macules, sometimes with a necrotic center, blisters, or larger necrolytic areas. Indeed dermoscopic structures correlate to histologic findings of SCARDS. Black dots and large necrotic areas under dermoscopy represent basal and suprabasal necrotic keratinocytes in early lesions and more extensive epidermal necrosis with subepidermal separation or vesiculation and even full-thickness epidermal necrosis in severe cases [31–35]. The black dots were present in all SCARDs, including DRESS, but also in some non-severe CADRs, like erythema multiforme and fixed drug eruption, where focal epidermal necrosis might also be found. Dermoscopy of erythema multiforme has already been described by Kalliyadan as red, blue, purple and black clods corresponding to the central dusky zone, a plain featureless area corresponding to the pale edematous zone, and homogenous erythema corresponding to the outer red ring [36]. A few short linear vessels were also described in this drug eruption [36]. The black dots are particularly helpful in the early detection of SCARDs because black dots are not seen clinically, and their identification together with the presence of systemic symptoms might raise the suspicion of a severe reaction earlier than clinical inspection with the naked eye alone [34,37].

**Figure 4:**
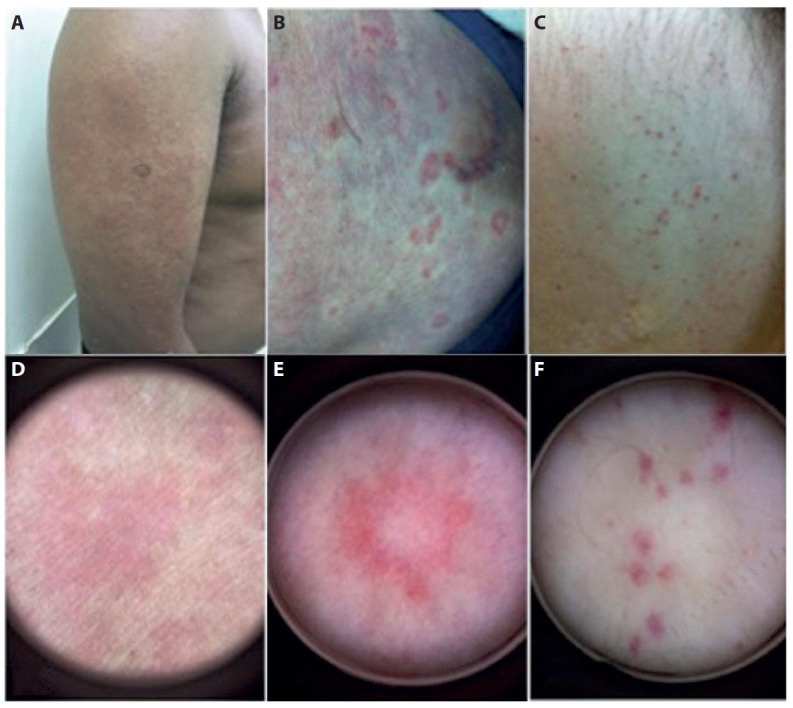
Clinical and dermoscopic images of non-severe cutaneous adverse drug reactions. (A) Clinical image demonstrating a patient with an exanthematous reaction and (D) showing erythema on dermoscopy. (B) Clinical image demonstrating a patient with urticaria and (E) showing erythema and linear vessels on dermoscopy. (C) Clinical image demonstrating a patient with cutaneous vasculitis and (F) showing purpuric dots (original magnification, ×10).

Exanthema was the most common reaction in our series. In these cases, dermoscopy was characterized mostly by the presence of erythema (Figure 4), purpuric dots and, less frequently, vascular structures. Errichetti et al. recently described an exanthematous drug eruption that presented a pinkish-reddish background and dotted/linear irregular vessels, but they did not describe purpuric dots [38]. In urticarial reactions, the same structures were seen, but purpuric dots were less prevalent than in exanthema (Figure 4). In drug-induced urticarial reactions, vasculitis may occur, but vessel involvement is usually subtle [32]. A report describing dermoscopic clues of common urticaria and urticaria vasculitis, revealed reticular red lines (corresponding to subpapillary vessels), in addition to structureless avascular areas representing prominent edema in common urticaria and purpuric dots or globules only in urticarial vasculitis [39].

In our study, the dermoscopic pattern of cutaneous vasculitis consisted mainly of purpuric dots (Figure 4). Dermoscopy of purpuric lesions has already been described. According to Vazquez-Lopez et al., leukocytoclastic vasculitis, is characterized by multiple small, speckled, blurred purpuric blotches or more defined purpuric globules over a purple, and later, orange-brown background [40].

When comparing the main dermoscopic structures in severe and non-severe reactions, black dots or necrotic areas, epidermal detachment, necrotic borders, scales and erosion correlated significantly with severe presentations (Table 2). Most studies that described dermoscopy of non-severe eruptions that may be related to drugs did not describe the presence of the black dots [41–44]. On the other hand, erythema, purpuric dots, and vascular structures did not differ between groups. Therefore, erythema, which was the most prevalent finding in both presentations, occurs in most cutaneous reactions and does not differentiate SCARDs from non-severe CADRs. It represents dilated blood vessels and inflammation, and a careful clinical and dermoscopic examination for other dermoscopic signs or structures should be conducted.

Our study has some limitations. The number of patients evaluated was limited to one year of data collection, and it may be that some non-severe CADRs were not included because our Department was not called. Also, we did not evaluate outpatients, which might have contributed to a greater proportion of severe cases. In addition, the median time from the onset of the cutaneous reaction declared by the patients and our evaluation was 5 days; consequently, early dermoscopic structures may have gone unnoticed in some cases, especially less severe ones.

## Conclusions

In conclusion, our data seem to indicate that dermoscopy helps to better characterize the different CADRs. SJS, TEN and overlap SJS/TEN lesions show black dots or necrotic areas, necrotic borders, erosion and epidermal detachment, while in the most common non-severe CADRs, erythema, purpuric dots, and vascular structures were commonly seen. Dermoscopy also improves the clinical recognition of SCARDs by detecting structures that are not visible with the naked eye and provides additional information to the clinicians. Beyond the scope of this study, future reports could evaluate if immediate dermoscopic evaluation of patients with suspicion of SCARDs might lead to changes in survival rates by dermoscopic detection of early signs of severity.

## Figures and Tables

**Table 1:** Frequency of Dermoscopic Structures in Different Presentations of CADRs

Dermoscopic structures	SJS n (%) 6 (8.7)	Overlap SJS/TEN n (%) 3 (4.3)	TEN n (%) 1 (1.4)	DRESS n (%) 6 (8.7)	Exanthem n (%) 33 (47.8)	Urticaria n (%) 6 (8.7)	EM n (%) 5 (7.2)	Cutaneous Vasculitis n (%) 4 (5.8)	Photosensitized Eczematous Reactions n (%) 2 (2.9)	Erythroderma n (%) 2 (2.9)	FDE n (%) 1 (1.4)
Erythema	5 (83.3)	3 (100.0)	–	5 (83.3)	31 (93.9)	6 (100.0)	4 (80.0)	–	2 (100.0)	2 (100.0)	1 (100.0)
Purpuric dots/purpura	3 (50.0)	1 (33.3)	–	4 (66.7)	24 (72.7)	2 (33.3)	4 (80.0)	4 (100.0)	1 (50.0)	–	–
Black dots/necrotic areas	6 (100.0)	3 (100.0)	1 (100.0)	2 (33.3)	–	–	2 (40.0)	–	–	–	1 (100.0)
Scales	2 (33.3)	1 (33.3)	–	2 (33.3)	2 (6.1)	–	–	–	1 (50.0)	2 (100.0)	–
Epidermal detachment	5 (83.3)	2 (66.7)	1 (100.0)	–	–	–	–	–	–	–	–
Vascular structures	–	–	–	1 (16.7)	11 (33.3)	3 (50.0)	1 (20.0)	–	–	–	1 (100.0)
Necrotic borders	4 (66.7)	3 (100.0)	1 (100.0)	–	–	–	–	–	–	–	–
Erosion	4 (66.7)	3 (100.0)	1 (100.0)	–	–	–	–	–	1 (50.0)	–	–

SJS = Stevens-Johnson syndrome; TEN = toxic epidermal necrolysis; DRESS = drug reaction with eosinophilia and systemic symptoms; EM = erythema multiforme; FDE = fixed drug eruption.

**Table 2:** Frequency of Dermoscopic Structures in Lesions of Severe and Non-Severe of Cutaneous Adverse Drug Reaction

	Severe* n=16 (23.2%)	Non-Severe n=53 (76.8%)	P value
Erythema	13 (81.2)	46 (86.8)	0.687
Purpuric dots	8 (50.0)	35 (66.0)	0.258
Black dots/necrotic areas	12 (75.0)	3 (5.7)	< 0.001
Scales	5 (31.2)	5 (9.4)	0.045
Epidermal detachment	8 (50.0)	–	< 0.001
Vascular structures	1 (6.2)	16 (30.2)	0.094
Necrotic borders	8 (50.0)	–	< 0.001
Erosion	8 (50.0)	1 (1.9)	< 0.001

* Toxic epidermal necrolysis, Stevens-Johnson syndrome, overlap Stevens-Johnson syndrome/toxic epidermal necrolysis, and reaction with eosinophilia and systemic symptoms
